# Ending the Epidemic: Assessing Sexual Health Communication, Personal Agency, and HIV Stigma among Black and Latino Youth in the U.S

**DOI:** 10.3390/ijerph18126319

**Published:** 2021-06-11

**Authors:** Lance Keene, Donte Boyd

**Affiliations:** 1 Silver School of Social Work, New York University, 1 Washington Square N., New York, NY 10003, USA; 2 College of Social Work, Ohio State University, 1947 College Rd N., Columbus, OH 43210, USA; boyd.465@osu.edu

**Keywords:** U.S. Black and Latino youth, sexual health communication, HIV stigma, personal agency, sexual health and HIV/STIs prevention

## Abstract

Sexual health communication warrants greater attention as it may help to reduce the rates of HIV incidence among youth. A growing body of literature suggests that conversations about sexual health among Black and Latino youth may serve as a potential strategy for HIV prevention. The current study investigates whether sexual health communication—in particular, conversations about sexual health and HIV—influences Black and Latino youth’s personal agency regarding their role in achieving an HIV-free generation. For this secondary data analysis, we used the National Survey of Teens and Young Adults on attitudes towards HIV/AIDS (*n* = 701). Participants included youth between the ages 15 and 24, and the average was 20 years. We used a multiple regression analysis to examine whether sexual health communication contributed to youth knowledge and awareness of (1) the national plan for EHE, and (2) their role in ending the epidemic”. (1) knowledge and awareness of the national plan for EHE, and (2) role in ending the epidemic. The final multiple regression model was statistically significant [R^2^ = 0.16 F (12, 701) = 001, *p* < 0.001] for both outcomes. Study results found that sexual health communication was positively related to Black and Latino youth’s awareness of efforts to end the HIV epidemic (EHE) and their belief that they could play a role in achieving EHE. In addition, HIV stigma influenced personal agency and whether youth were aware of efforts to achieve EHE. Our results demonstrated that openly communicating about sexual health and HIV may contribute to a sense of personal agency among Black and Latino youth. In addition, understanding whether sexual health communication contributes to a sense of personal agency among youth may inform HIV prevention efforts to achieve the goals set forth by the national EHE plan for the U.S.

## 1. Introduction

The United States (U.S.) federal government has established a goal of ending the HIV epidemic (EHE) by 2030 [1]. Despite reductions in rates of HIV incidence, the virus increasingly affects younger adolescents and young adults [2]. Youth aged 13 to 24 comprise 20% of new HIV diagnoses, although many are likely unaware of their diagnosis [3]. Moreover, racial disparities pertaining to HIV incidence are widely documented [4], whereby racial minority populations, such as Black and Latino Americans, remain disproportionately impacted [5]. U.S. Black and Latino youth, in particular, currently report the highest rates of HIV incidence [5], with Black youth accounting for more than 60% of new HIV infections when compared to their Latino (20%) and White (20%) counterparts [5]. Beyond the disproportionate impact on U.S. Black communities, recent studies document an escalating impact on U.S. Latino populations. A recent study addressing the “invisible epidemic” among U.S. Latinos demonstrates that HIV incidence has increased in Latino communities by more than 14% since 2010 [6]. Additionally, while much HIV literature emphasizes its disproportionate impact on diverse populations of men (e.g., sexual minority men, heterosexual men), increasing rates of HIV incidence have been observed across gender. Indeed, a growing trend of HIV incidence among increasingly younger Black and Latino youth, male and female [6,7,8,9,10], demonstrates the need for youth-focused approaches to HIV prevention. National efforts to curtail rising HIV incidence rates require perspectives that emphasize agency among Black and Latino youth. Presently, national health promotion strategies encourage reductions in HIV incidence by encouraging individuals at risk of HIV to use a range of proven effective prevention strategies (e.g., consistent and correct condom use, limiting number of sexual partners, and uptake of pre-exposure prophylaxis) [11]. To the authors’ knowledge, no current national strategies focus on sexual health communication among adolescents and young adults as a strategy for HIV prevention. In addition, national efforts should emphasize the importance of prevention and control of sexually transmitted infections (STIs) among adolescents and young adults which also may reduce HIV incidence [12]. For instance, cervical infection can also be a concern due to high prevalence among adolescents and young adults, potentially leading to heightened risk of HIV transmission [12]. In this study, we examine whether sexual health communication contributes to youth knowledge and awareness of (1) the national plan for EHE, and (2) their role in ending the epidemic”. 

### 1.1. Black and Latino Youth in the U.S.

Black and Latino youth in the U.S. experience stigma, discrimination, and racism [13,14,15,16]. As a result, many navigate social inequities and exclusions that place them at greater risk for HIV (e.g., poverty, limited access to quality healthcare, psychosocial stress) [15,16,17]. Presently, Black and Latino youth in the U.S. are more likely to report living in lower socioeconomic communities [18], lower rates of educational attainment [19,20], and limited access to quality healthcare, including for sexual and reproductive health [21]. Collectively, these factors place Black and Latino youth at heightened vulnerability to HIV [15,17]. Additionally, HIV-related stigma remains a major barrier influencing HIV prevention in the U.S. Beyond serving as a major barrier to accessing prevention, care, and treatment services, HIV-related stigma has also been cited as limiting critical conversations about sexual health and HIV prevention [22]. In addition, Black and Latino youth populations may encounter stigmas that are interrelated with their racial, gender, and/or sexual identities [23,24,25,26]. Intersectional stigma, for instance, describes the overlapping stigma associated with race, gender, and sexual orientation that disproportionately impacts Black and Latino populations [27,28]. Adolescent and young adult Black and Latino youth may encounter racial, gender, and sexual stereotyping that overlap with existing societal stigmas about HIV which may also negatively impact sexual health communication [23,24,25,26,27,28]. National efforts that seek to reduce HIV among both communities should consider the ongoing influence of HIV-related stigma in sexual health communication among key populations, e.g., Black and Latino adolescents and young adults. 

### 1.2. Sexual Behavior among Adolescents and Young Adults 

Extant research indicates that high-risk sexual contact remains the most common method of HIV transmission among youth [29]. Sexual risk-taking has been defined in multiple ways, including engaging in unprotected sex, having multiple sexual partners, and early age of sexual debut—behaviors that have consistently been shown to heighten individuals’ risk of acquiring HIV and other STIs [29,30]. According to the CDC’s 2019 Youth Risk Behavior Survey (YRBS), which assessed a range of health-risk behaviors (e.g., sexual behavior, high-risk substance use, experiencing violence, and mental health and suicide), among 9th to 12th grade students nationally [31], 38.4% of adolescents surveyed reported having sexual intercourse at least once, while approximately 8.6% of adolescents reported four or more lifetime sexual partners. Additionally, 45.7% of adolescents had failed to use condoms during their last sexual encounter [31]. Although research indicates that the majority of adolescents report condom use during their last sexual encounter, many often fail to use condoms consistently, increasing their likelihood of HIV acquisition [32,33].

Of concern is that despite advancements in HIV prevention and treatment, HIV incidence rates remain high among adolescents [32], suggesting that new approaches to HIV prevention with youth are needed. To date, the majority of HIV prevention education within school- and community-based settings targeting adolescents has been focused on addressing individual-level health promotion including consistent condom use and limiting the number of sexual partners, behaviors which are important for HIV prevention [33,34]. However, studies also suggest that youth may not perceive themselves to be at risk for HIV and may underestimate their risk on the basis of their reported sexual behavior (i.e., condom use, number of sexual partners, or partner risk) [35]. Additionally, youth may also engage in behaviors that increase their likelihood for HIV acquisition despite an awareness of the risks involved [35]. Taken together, these findings suggest that additional youth-focused HIV prevention strategies are needed. 

### 1.3. Sexual Health Communication

Sexual health communication warrants greater attention as it may help to reduce rates of HIV incidence among youth. A growing body of literature suggests that sexual health communication among Black and Latino youth may serve as a potential strategy for HIV prevention. For instance, Mullinax and colleagues recently found that adolescents who received information about sexual health from a personal source (e.g., peers and sexual partners) increased their HIV testing [36]. Additionally, another recent study found that youth who discuss a range of sexual health topics (e.g., condom use, HIV prevention, and sexually transmitted infections) with either their peers or parents were more likely to use condoms and to limit their casual sexual encounters [37]. Despite a relatively small body of scholarship, recent studies of sexual health communication focusing on racial minority youth suggest that such communication may serve as an effective HIV prevention strategy, given that the information that is shared is derived from influential network members within Black and Latino youth social networks (e.g., parents, peers, romantic partners) [38,39]. Therefore, it is possible that sexual health information shared among peers, for instance, may be viewed more favorably, in turn, contributing to strengthening youth’s desire to maintain HIV-negative status. 

### 1.4. Personal Agency

Considerable research investigates the role of personal agency in maintaining health [40,41,42,43]. Personal agency has been defined in multiple ways (e.g., perceived behavioral control, self-efficacy, and perceived power) and is considered a determinant of individual behavioral intention and subsequent health outcomes [40,41,42,43]. Within behavioral health contexts, personal agency is often conceptualized as an individuals’ perceived control to achieve a desired health outcome [40,41,42,43]. However, a range of factors, including perceived severity of illness (e.g., HIV) may influence individuals’ sense of personal agency relating to HIV prevention [44]. For Black and Latino adolescents and young adults in the U.S., recognition of the potential impact of HIV on physical health and enduring stigma related to HIV may foster a sense of personal agency among youth whose communities remain disproportionately impacted [45,46]. Overall, within the context of recent studies that focus on HIV prevention, the concept of personal agency has been demonstrated to inform proactive sexual health approaches among youth, potentially reducing their sexual risk behavior and exposure to HIV.

### 1.5. Present Study

To date, few studies of Black and Latino youth populations have investigated the role of sexual health communication as a potential strategy for curtailing HIV incidence. The current study investigates whether sexual health communication informs a sense of personal agency among Black and Latino youth regarding their sexual health to achieve an HIV-free generation. In this study, we examined whether sexual health communication contributed to youth knowledge and awareness of (1) the national plan for EHE, and (2) their role in ending the epidemic”.. Understanding whether sexual health communication contributes to a sense of personal agency among Black and Latino youth may inform HIV prevention efforts and help to achieve the goals set forth by the national plan. 

## 2. Materials and Methods

Designed and implemented by public opinion researchers at the Kaiser Family Foundation, the National Survey of Teens and Young Adults on HIV/AIDS assessed the knowledge, stigma, beliefs, and comfort of youth and young adults around HIV. The 40-question, web-based survey was conducted with 1437 youth (aged 15–24) from 21 September 2012 to 1 October 2012. The respondents of the survey were members of the Knowledge Panel, a randomly drawn representative national panel of households that were selected using address-based sampling methods to participate by telephone. Knowledge Panel surveys use a dual sampling method that includes households with (1) listed phone numbers, (2) unlisted phone numbers, (3) telephones, (4) no telephone, and (5) only cell phone access. The participants completed self-administered mail and web surveys, and households were provided with technology to access the internet, if necessary. This differed from other forms of internet research that included only individuals who could already access the internet. 

Due to the sensitive subject matter, the parents of those participants aged 15–17 were provided a summary of the survey and had to give consent for their children to participate. Of the total number of youths contacted, 77% of parents allowed their children to participate. The data were weighted to balance the sample demographics with estimates of the national population collected by the Census Bureau in August 2012. The present study was conducted using secondary data from the National Survey of Teens and Young Adults on HIV/AIDS, which are freely available on request, therefore, no ethical approval or consent was needed. 

### 2.1. Measures

The two outcome variables of this study pertaining to personal agency included role in ending the epidemic and having heard about ending the epidemic. Role in ending the epidemic was measured by the following question: “How much of a role, if any, do you think you personally can play in achieving the goal of an AIDS-free generation?” This was a single-item, four-point response scale (ranging from 1 = I don’t want to play a role to 4 = A big role) with a higher score indicating a greater belief in the ability to play a role in helping to end the epidemic, the overall mean score for this item was 2.73 (SD = 0.92). Having heard about ending the epidemic was measured by the following question: “Recently, there has been discussion about the possibility of achieving an AIDS-free generation. How much, if anything, have you heard about the goal of an AIDS-free generation?” This was a single-item, four-point response scale (ranging from 1 = Nothing at all to 4 = A lot) with a higher score indicating having heard more about an AIDS-free generation, the overall mean score for this item was 1.69 (SD = 0.97) [47,48]. 

Several contextual variables were collected. Sexual health communication was measured by a single-item, four-point response scale (ranging from 1 = Never to 4 = Often) asking whether the respondents had held a conversation with someone about HIV/AIDS or other STIs in the past year. The overall mean for this item was 1.96 (SD = 0.91). Glad the person brought it up was measured by a single-item, four-point response scale (ranging from 1 = Strongly disagree to 4 = Strongly agree) asking respondents, “If someone you were seeing romantically suggested that you get tested together for HIV, would you be glad the person brought it up?” A higher score indicated greater agreement. The overall mean for this item was 1.84 (SD = 0.82) [48]. 

The following independent variables are a series of single items, were evaluated on a two-point response scale (ranging from 0 = No, would not to 1 = Yes, would like more information) and asked respondents the following questions: “Please tell me whether (or not) this is something you would like more information about: How to talk to a partner about getting tested for STDs (including HIV)”, (*M*= 1.35, *SD* = 0.47) and “How to talk to a partner about using condoms”, (*M* = 1.27, *SD* = 0.44).

The following independent variables, a series of single items, were evaluated on a two-point response scale (ranging from 0 = No to 1 = Yes) and asked respondents the following questions: “Thinking about your current or most recent sexual relationship, have you ever talked to your partner about HIV and condoms?” (*M* = 1.38, *SD* = 0.48; and *M* = 1.80, *SD* = 0.39, respectvely.

Moreover, the HIV testing status, in this study, was based on the participants’ responses to the following: “Have you, yourself, ever been tested for HIV?” The responses were coded 0 for a negative response and 1 for an affirmative response.

HIV stigma was measured using a single-item, four-point response scale (ranging from 1 = A lot to 4 = None at all) and asked respondents the following question: “How much stigma, if any, do you think there is in the United States today around HIV/AIDS?” The overall mean for this item was 1.82 (SD = 0.71).

Sociodemographic characteristics were also collected. Participants were asked to indicate their age, race and ethnicity, gender, sexual orientation, household income, education attainment, and region of the country. Age and household income are continuous variables. Gender was coded 0 if male and 1 if female. Race and ethnicity was coded as 1 = White, 2 = Black, 3 = Hispanic, 4 = other, on-Hispanic, and 5 = two or more races. Sexual orientation was coded as 1 = heterosexual, 2 = gay, 3 = lesbian, 4 = bisexual, and 5 = other. Education attainment was coded 1 = less than high school, 2 = high school diploma, 3 = some college, and 4 = bachelor’s degree or higher. Lastly, region of the country was coded as 1 = Northeast, 2 = Midwest, 3 = South, and 4 = West. 

### 2.2. Data Analysis

All analyses were conducted on observations that included non-missing data for both outcomes, role in ending the epidemic and having heard about ending the epidemic. Statistical tests of association were conducted between the measures described in Section 2.2. and the two outcomes. Table 1 provides descriptive statistics for the study sample. Table 2 provides a bivariate correlation analysis for all the predictor variables—sexual health communication, glad the person brought it up, wanting information on talking to a partner about HIV testing and using condoms, having talked with a sexual partner about HIV and condom, HIV testing, HIV stigma—concerning age, household income, education attainment, and the outcome variable: personal agency (role in EHE and having heard about EHE). Table 3 presents a multivariate analysis regression model that examined the predictor variables: sexual health communication, glad the person brought it up, wanting information on talking to a partner about HIV testing and using condoms, having talked with a sexual partner about HIV and condom, HIV testing, HIV stigma, the accompanying covariates (gender, age, race and ethnicity, household income, region, sexual orientation and education attainment), and the outcome variable—having heard about ending the epidemic. Table 4 presents aforementioned predictors, covariates, and the outcome variable role in ending the epidemic. For survey scales, a mean score of the scale items was generated for participants with non-missing data. All analyses were conducted using STATA 17.

## 3. Results

### 3.1. Sample Characteristics 

As noted in Table 1, the total sample was *n* = 1437 and most of the participants self-identified as female (56%). The average age was 20 years (SD = 3.02). A majority of the youth and young adults self-identified as heterosexual (91%). A majority of the sample was between the ages of 18 and 24 (61%), and the average household income was between USD 35,000 and USD 39,000. Additionally, roughly 59% of the participants (*n* = 412) reported that they were sexually active during the study period and 66% (455) had not received an HIV test. A majority of participants reported living the US South (35%) and a majority reported having some college (43%). 

#### 3.1.1. Bivariate Analysis

All the variables in the bivariate correlation analysis indicated a relationship between the predictor variables and the outcome: personal agency. For instance, our results indicate that there was a significant positive association between sexual health communication and personal agency in playing a role in EHE (*r* = 0.27, *p* < 0.01). A positive correlation was observed between (1) wanting information on talking about HIV (*r* = 0.21, *p* < 0.01) and (2) condoms (*r* = 0.23, *p* < 0.01) on playing a role in EHE. 

#### 3.1.2. Multiple Regression Analyses

Table 3 presents study predictors, covariates, and the outcome variable: *having heard about EHE*. The model only included youth and young adults who reported being sexually active. The overall model was statistically significant [R^2^ = 0.16 F (16, 701) = 000, *p* < 0.001]. Our results indicated a statistically significant and positive relationship between sexual health communication and having heard about EHE (β = 0.19; *p* < 0.001). Surprisingly, our results indicated a negative relationship between wanting information on talking about HIV and having heard about EHE (β = −0.11; *p* < 0.01) and a positive relationship between wanting information on talking about condoms and having heard about EHE (β = 0.21; *p* < 0.001). There was also a positive relationship between communicating with your sexual partner about condoms and having heard about EHE (β = 0.15; *p* < 0.01). HIV stigma had a negative relationship with youth and young adults having heard about EHE (β = −0.12; *p* < 0.001). Living in the Midwest was statistically significantly negatively associated with having heard about EHE (β = −0.9; *p* <.001). Lastly, having some college was negatively associated with having heard about EHE (β= −0.18; *p* < 0.001). 

Table 4 presents study predictors, covariates, and the outcome variable: *role in ending the epidemic*. The overall model was statistically significant [R^2^ = 0.21 F (16, 701) = 000, *p* < 0.001]. The model only included youth and young adults who reported being sexually active. The results indicated a positive relationship between sexual health communication and playing a role in EHE (β = 0.13; *p* < 0.01). Additionally, we observed a positive relationship between wanting information on talking about condoms and playing a role in EHE (β = 0.14; *p* < 0.01). Our results also indicated a positive relationship between being glad the person brought it up and playing a role in EHE (β = 0.14; *p* < 0.001). HIV stigma had a negative relationship with youth and young adults in their role EHE (β = −0.12; *p* < 0.001). Those who self-identified as Black (β = 0.08; *p* < 0.05), and two or more races, non-Hispanic (β = 0.07; *p* < 0.05) were positively associated with believing they can play a role in achieving EHE. Lastly, having a bachelor’s degree or higher was positively associated with belief that individuals can play a role in achieving EHE (β = 0.14; *p* < 0.001). 

## 4. Discussion

Sexual health communication is associated with safer sexual decision-making [49], decreased risk of HIV transmission [50], and engaging in fewer risk behaviors [29]. Prior studies have demonstrated that sexual health communication can inform a sense of agency among youth in maintaining their sexual health [51]. Given that adolescents and young adults continue to be disproportionately impacted by HIV [49], achieving the goals set forth by the national plan for EHE requires integrating youth-focused approaches, emphasizing personal agency and self-efficacy, while highlighting the important role of communication among influential network members (e.g., peers, sexual partners) surrounding the maintenance of sexual health (e.g., HIV testing, condom use). Given that Black and Latino youth in the U.S. are disproportionately affected by HIV and in need of culturally and developmentally tailored HIV prevention, consideration should be given to the role that culture plays in information dissemination and retention. Recent studies suggest that national and local efforts that incorporate key cultural characteristics such as language and context are particularly effective with young people, and potentially effective in reducing high risk sexual behaviors and increasing HIV testing [52,53,54]. Additionally, beyond basic health literacy, strengthening individuals interactive health literacy may reduce disparities, promote greater health equity and help to achieve EHE by strengthening individuals’ ability to discuss safer sex negotiation with their peers and sexual partners as well as strengthen their interactions with healthcare providers [55,56]. Finally, to achieve the goals of EHE, in addition to strengthening peer education (i.e., amongst peers and/or sexual partners), greater attentiveness to the dissemination of information regarding sexual health should be integrated into secondary and post-secondary educational contexts as well, as extant studies demonstrate their effectiveness in educational settings [57,58]. In sum, to complement the national plan’s current focus to diagnose, treat, prevent, and respond [59], greater attentiveness to how sexual health communication contributes to a sense of personal agency among Black and Latino youth may inform the development of future culturally tailored HIV prevention efforts to suit their respective needs.

In the present study, the main finding is that sexual health communication among Black and Latino adolescents and young adults was positively related to believing that they can personally play a role in achieving EHE. This finding and the additional results discussed can be understood in light of perspectives on personal agency [60,61]. In the sample of Black and Latino youth that was included in the study, we found that having conversations with peers and/or sexual partners about sexual health was positively related to youth believing that they can personally play a role in EHE. For instance, we found that having had a conversation with someone about HIV/AIDS or other STIs in the past year and being glad that a sexual partner brought up getting tested together for HIV were both related to youth believing that they personally can contribute to EHE. Prior studies have described the important role that influential network members, such as peers and sexual partners, play in contributing to the sexual health decision-making of adolescents and young adults [44,45,48]. For example, not only does openly communicating about sexual health issues with a sexual partner encourage safer sexual decision-making throughout adolescence and young adulthood, but prior studies demonstrate that youth who engage in more frequent sexual health communication with their partners were more likely to delay sexual debut and to use condoms more consistently once they began engaging in regular sexual intercourse [50,61,62]. In addition to influencing youth’s sexual health decision-making, their sense of personal agency and sexual self-efficacy are strengthened by having conversations with influential network members (e.g., peers, romantic and sexual partners) about sexual health [62]. Beyond contributing to a greater capacity among youth to demonstrate sexual assertiveness in relation to safe sex in their sexual relationships [62], open communication about sexual health may instill a sense in youth that they can personally contribute to achieving EHE.

Notably, HIV stigma was negatively related to Black and Latino youth’s believing that they personally can play a role in EHE. This finding aligns with results from prior research on HIV stigma [62]. HIV stigma has been found to impede critical conversations about sexual health, HIV, and other STIs [62]. For adolescents, HIV stigma may be heightened given limited sexual experience and limited information available relating to sexual health [62]. In addition, this finding also suggests that national EHE efforts must continue to highlight and address HIV stigma in order to make progress toward national goals.

Interestingly, we also identified positive associations between youth’s racial identities—e.g., Black non-Hispanic and two or more races non-Hispanic—and believing that youth can personally play a role in EHE. An interpretation of this finding is that some racial minority youth (e.g., Black adolescents and young adults) may possess a heightened awareness of the disproportionate rates of HIV and other STIs within their communities and, therefore, may be attentive to ensuring that they play a role in achieving EHE. Notably, Hispanic identity was not positively related to contributing to EHE. This finding may suggest that Hispanic youth do not perceive themselves as being at similar levels of risk for HIV as other racial minorities (e.g., Black adolescents and young adults). However, given the rising HIV incidence rates among U.S. Latino communities, it is important to highlight the HIV vulnerability of at-risk Latino populations as part of ongoing national efforts to achieve EHE. Finally, possessing a bachelor’s degree or higher was also related to individuals’ believing that they personally can play a role in achieving EHE. This finding suggests that greater educational attainment may be associated with greater sense of self-efficacy in relation to one’s sexual health.

Another main finding is that sexual health communication was related to having heard about ending the epidemic. Specifically, we found that having had a conversation with a sexual partner about HIV and having had a conversation with a sexual partner about condom were positively related to having heard about EHE. One interpretation of this finding is that youth’s awareness of the efforts to achieve EHE may encourage more open discussions of HIV and condoms in their romantic and sexual relationships. Therefore, increasing Black and Latino adolescents and young adults’ awareness of the ongoing national effort to achieve EHE is critical. Additionally, wanting information on talking about HIV was negatively related to having heard about EHE, while wanting information about condoms, conversely, was positively related to having heard about national EHE efforts. This finding suggests that youth desiring additional information about HIV were less aware of national efforts to achieve EHE than those desiring information about condoms. Again, this finding highlights the critical importance of raising awareness of EHE efforts within communities, especially in relation to HIV. HIV stigma was negatively related to having heard about EHE. Again, this finding highlights the critical role of HIV stigma in relation to sexual health promotion and provides further justification for highlighting the influence of HIV stigma on national HIV prevention efforts. Finally, the negative relationships between living in the Midwest, completing some college education, and individuals’ awareness of EHE, suggest that some demographics are relatively more advantaged (e.g., living in the Northeast, having more education) than others in relation to having heard about EHE efforts.

Our results should be interpreted in light of study limitations, including use of a cross-sectional research design and self-report data. Despite these limitations, our study results expand the current literature by gaining insight into the influence of sexual health communication and personal agency in safer sex negotiation among Black and Latino youth. Future research into adolescent and young adult sexual health should consider how sexual health communication interventions can improve sexual health and wellness, including HIV/STIs prevention, among similarly aged sexual and gender minorities in Black and Latino communities, particularly given the disproportionate rates of HIV incidence within these communities.

## 5. Conclusions

Overall, our investigation found that sexual health communication among Black and Latino youth informed their sense of personal agency to end the HIV epidemic. Additionally, the study reaffirmed the role of influential network members (e.g., youth’s peers and sexual partners) in the discussion of sexual health topics (e.g., conversations about HIV, HIV testing, and condoms) and sexual health decision-making (e.g., wanting information on discussing using condoms and testing with a sexual partner) among youth. Moreover, the study demonstrates youth’s awareness of national strategies to end the epidemic and suggests that youth receive sexual health information from multiple sources and that open communication about sexual health with their peers and sexual partners plays a key role in their sexual decision-making and subsequent sexual health outcomes.

Study findings inform several key recommendations and strategies for optimizing sexual health and well-being among U.S. Black and Latino youth. Given that youth are embedded within multiple social contexts, these recommendations are designed to inform a multilevel intervention focused at the interpersonal, community, and national level. First, at the interpersonal level, development and optimization of peer-led interventions that are focused on strengthening individual Black and Latino adolescents’ and young adults’ self-efficacy to communicate with their peers and sexual partners about sexual health topics including sex, HIV and HIV testing, and correct and consistent condom use, may increase individual youth’s sense of personal agency to curtail HIV incidence rates. At the community level, evidence-based initiatives in schools and community-based organizations that provide youth with culturally responsive sexual health information and decision-making strategies may enhance youth’s collective sense of empowerment around maintaining their sexual health. Finally, at the national level, in conjunction with the national plan for ending the epidemic, allocating additional federal funds to increase the number of local, regional, and national organizations and institutions (e.g., schools and community-based organizations) that design and implement sexual health and HIV prevention curriculum to youth.

## Figures and Tables

**Table 1:** Sample characteristics (*n* = 1437).

Variables	Frequency	Percentage
Race and Ethnicity		
African American or Black	271	39
Hispanic	322	46
Other, Non-Hispanic	57	8.0
Two+ Races, Non-Hispanic	51	7.2
Gender		
Male	312	44
Female	392	57
Sexual Orientation		
Heterosexual	1308	92
Gay	16	1.13
Lesbian	13	1.00
Bisexual	53	4.00
Other	26	1.84
Household Income		
USD 29,000 or less	328	47
USD 30,000–USD 59,000	205	29
USD 60,000–USD 84,999	71	10
USD 85,000–USD 99,000	33	5
USD 100,000 and above	67	10
Education Attainment		
Less than high School	134	9
High School	399	28
Some College	616	43
Bachelor’s Degree or higher	288	20
HIV Testing		
Yes	238	66
No	455	34
Sexually Active		
Yes	412	59
No	267	38
Refused	25	4.0
Region of Country		
Northeast	237	16
Midwest	344	24
South	491	34
West	365	25

**Table 2:** Bivariate correlations, means, and standard deviations of the study variables (*n* = 701).

Variables	1	2	3	4	5	6	7	8	9	10	11	12
1. Role in ending the epidemic	1.000											
2. Heard about ending the epidemic	0.22 *	1.000										
3. Sexual health communication	0.27 ***	0.24 ***	1.000									
4. Glad the person brought it up	−0.22 ***	0.07 *	−0.24 ***	1.000								
5. Information on talking about HIV	0.21 ***	0.14 ***	0.20 ***	−0.16 ***	1.000							
6. Information on talking about condoms	0.23 ***	0.19 ***	0.18 ***	−0.14 ***	0.72 ***	1.000						
7. Conversation with sexual partner about condoms	0.27 ***	0.24 ***	0.15 ***	−0.24 ***	0.19 ***	0.18 ***	1.000					
8. Conversation with sexual partner about HIV	0.16 ***	0.17 ***	0.45 ***	−0.23 ***	0.07 *	0.05	0.45 ***	1.000				
9. HIV Testing	0.11 ***	0.15 ***	0.28 ***	−0.15 ***	0.05	0.04	0.28 ***	0.32 ***	1.000			
10.HIV Stigma	−0.23 ***	−0.20 ***	−0.20 ***	0.15 ***	−0.12 ***	−0.10 ***	−.20 ***	−0.13 **	−0.03 *	1.000		
11.Age	−0.04	0.02	0.01	−0.04	−0.11 ***	−0.12 ***	0.01	0.13 ***	0.34 ***	0.01	1.000	
12. Household Income	−0.07 **	−0.16 ***	0.15 ***	0.10 **	0.08 *	−0.10 **	−0.14 ***	−0.10 **	0.24 **	0.06 *	−0.15 ***	1.000
13. Education Attainment	0.06	−0.12 ***	−0.01	−0.01	−0.05 *	−0.07 *	−0.01	0.06	−0.07 *	0.01	0.08 *	0.34 ***

*p* < 0.05 *, *p* < 0.01 **, *p* < 0.001 ***.

**Table 3:** Multivariate analysis among sexually active youth and young adults on having heard about ending the epidemic (*n* = 701).

Having heard about Ending the Epidemic	B	SE	β
Sexual Health Communication	0.19 ***	0.04	0.18 ***
Information on talking about HIV	−0.23 **	0.10	−0.11 **
Information on talking about condoms	0.48 ***	0.11	0.21 ***
Conversation with sexual partner about HIV	−0.09	0.10	−0.08
Conversation with sexual partner about condoms	0.31 **	0.18	0.15 **
Brought up HIV	0.00	0.05	0.00
HIV Testing	0.06	0.08	0.03
HIV stigma	−0.16 ***	0.05	−0.12 ***
Race and Ethnicity (White reference)			
Black, Non-Hispanic	−0.16	0.10	−0.07
Other, Non-Hispanic	0.17	0.18	0.03
Hispanic	−0.06	0.10	−0.03
Two+ Races, Non-Hispanic	−0.25	0.20	−0.04
Gender (Female reference)			
Male	0.01	0.07	0.00
Age	0.00	0.02	−0.01
Household Income	−0.01	0.01	−0.05
Region (Northeast reference)			
Midwest	−0.20 ***	0.11	−0.09 ***
South	−0.06	0.10	−0.03
West	−0.07	0.12	−0.03
Sexual Orientation (heterosexual reference)			
Gay	0.02	0.28	0.00
Lesbian	0.24	0.30	0.03
Bisexual	0.25	0.16	0.05
Other, please specify	−0.04	0.32	0.00
Education (less than high school reference)			
High school	−0.20	0.14	−0.09
Some college	−0.35 ***	0.13	−0.18 ***
Bachelor’s degree or higher	−0.23	0.16	−0.09

*p* < 0.01 **, *p* < 0.001 ***; B, unstandardized coefficient; SE, standardized errors; β standardized errors.

**Table 4:** Multivariate Analysis among sexually active youth and young adults on role in ending the epidemic (*n* = 701).

Role in Ending the Epidemic	B	SE	β
Sexual Health Communication	0.12 **	0.03	0.13 **
Information on talking about HIV	0.03	0.08	0.02
Information on talking about condoms	0.27 **	0.11	0.14 **
Conversation with sexual partner about HIV	−0.10	0.08	−0.10
Conversation with sexual partner about condoms	0.20	0.15	0.11
Brought up HIV	0.15 ***	0.04	0.14 ***
HIV Testing	−0.07	0.07	0.03
HIV stigma	−0.16 ***	0.05	−0.12 ***
Race and Ethnicity (White reference)			
Black, Non-Hispanic	0.16 *	0.08	0.08 *
Other, Non-Hispanic	0.10	0.16	0.02
Hispanic	0.16	0.08	0.07
Two+ Races, Non-Hispanic	0.34 *	0.17	0.07 *
Gender (Female reference)			
Male	0.08	0.06	0.04
Age	−0.01	0.01	−0.03
Household Income	0.00	0.01	0.00
Region (Northeast reference)			
Midwest	−0.05	0.10	−0.02
South	0.02	0.09	0.01
West	0.07	0.10	0.03
Sexual Orientation (heterosexual reference)			
Gay	0.16	0.24	0.02
Lesbian	0.23	0.25	0.03
Bisexual	−0.07	0.14	−0.02
Other, please specify	−0.01	0.27	−0.00
Education (less than high school reference)			
High school	0.17	0.12	0.09
Some college	0.16	0.11	0.08
Bachelor’s degree or higher	0.32 ***	0.13	0.14 ***

*p* < 0.05 *, *p* < 0.01 **, *p* < 0.001 ***; B, unstandardized coefficient; SE, standardized errors; β standardized errors.

## Data Availability

The present study was conducted using secondary data from the National Survey of Teens and Young Adults on HIV/AIDS, which are freely available on request.
